# Modulation of Tumor-Associated Macrophages to Overcome Immune Suppression in the Hepatocellular Carcinoma Microenvironment

**DOI:** 10.3390/cancers17010066

**Published:** 2024-12-29

**Authors:** Mahmoud Singer, Zhuoli Zhang, Farshid Dayyani, Zigeng Zhang, Vahid Yaghmai, April Choi, Jennifer Valerin, David Imagawa, Nadine Abi-Jaoudeh

**Affiliations:** 1 Department of Radiological Sciences, School of Medicine, University of California, Irvine, CA 92617, USA; zhuoliz1@hs.uci.edu (Z.Z.); zigengz@hs.uci.edu (Z.Z.); vyaghmai@hs.uci.edu (V.Y.); 2 Department of Medicine, Chao Family Comprehensive Cancer Center, University of California, Irvine, CA 92867, USA; fdayyani@hs.uci.edu (F.D.); aprilc@hs.uci.edu (A.C.); jvalerin@hs.uci.edu (J.V.); 3 Department of Surgery, University of California Irvine, Orange, CA 92697, USA; dkimagaw@hs.uci.edu

**Keywords:** tumor-associated macrophages, macrophages, TAMs, depletion, immunotherapy, HCC, liver cancer, tumor microenvironment, suppression TME

## Abstract

Hepatocellular carcinoma (HCC) is a major global health issue characterized by poor prognosis and complex tumor biology. One of the critical components of the HCC tumor microenvironment (TME) is tumor-associated macrophages (TAMs), which play a pivotal role in modulating tumor growth, immune evasion, and metastasis. Macrophages are divided into two major subtypes: pro-inflammatory M1 and anti-inflammatory M2, both of which may exist in TME with altered function and proportion. The anti-inflammatory M2 macrophages are further subdivided into four distinct immune suppressive subsets. TAMs are generally counted as M2-like macrophages with altered immune suppressive functions that exert a significant influence on both cancer progression and the ability of tumors to escape immune surveillance. Their involvement in modulating immune responses via different mechanisms at the local and systemic levels has made them a key target for therapeutic interventions seeking to enhance treatment outcomes. How TAMs’ depletion influences immune responses in cancer is the primary interest in cancer immunotherapies. The purpose of this review is to delve into the recent progress made in TAM-targeting therapies. We will explore the current theories, benefits, and challenges associated with TAMs’ depletion or inhibition. The manuscript concludes with future directions and potential implications for clinical practice.

## Simple Summary

Tumor-associated macrophages (TAMs) represent a challenge in developing an effective and safe therapy for HCC. TAMs are considered to be one of the major mechanisms for HCC resistance to current therapies. TAMs, known as M1-like and M2-like macrophages, were discovered to be subdivided into four subsets (M2a, M2b, M2c, and M2d). Each subset has a hypothetical contribution as a TAM immunosuppressive cell. Several trials have studied removing or modulating the immune suppressive roles of TAMs in the HCC microenvironment. Identifying TAMs’ behavior and subsets may be crucial for modulating the immune suppression in the HCC microenvironment.

## 1. Introduction

Hepatocellular carcinoma (HCC) accounts for 75–85% of primary liver cancer instances and ranks as the third highest cause of yearly cancer fatalities globally, with 41,210 newly diagnosed cases of liver cancer and an estimated death toll of 29,380. The outlook for patients with HCC is still unfavorable because of late detection, the immune tolerant nature of the liver, the aggressiveness of HCC, and the interplay between tumor cells and the tumor microenvironment (TME) [1,2]. One of the critical elements of the TME is the population of tumor-associated macrophages (TAMs) [3]. Most TAMs predominately possess immune suppressive functions and phenotypically are matched with M2 macrophages. The majority of TAMs fall under the category of M1-like and M2-like macrophages, indicating their alignment with the M2-like macrophage subtype that fosters tumor growth and suppresses immune reactions [4,5].

TAMs are macrophages derived from monocytes that are attracted to tumor tissues by chemokines present in the TME [6]. TAMs show a high immune suppression capacity by promoting tumor proliferation, drug resistance, and immune escape, as well as by enhancing tumor cell invasion, metastasis, and epithelial-mesenchymal transition (EMT) [5]. Macrophages can polarize differently into M1 and M2, which display opposite effects. An imbalance between M1 and M2 macrophages is connected to various diseases [7]. The presence of TAMs plays a significant role in the progression, metastasis, and development of therapeutic resistance in HCC. Recent treatment strategies have targeted TAMs [8], with clinical trials attempting to reprogram macrophages in the tumor microenvironment to complement existing therapies. This article will focus on exploring the role of TAMs in HCC, assessing the existing methods for targeting TAMs, addressing the potential benefits and obstacles associated with these strategies, and exploring potential future directions.

## 2. The Role of TAMs in Cancers

### 2.1. Macrophage Biology and Subtypes

Macrophages originate from circulating monocytes that transform into M1 and/or M2 macrophages when they migrate from the bloodstream to different organs [9,10]. In cancers, monocytes transform at tumor sites in response to tumor-derived signals [11]. There are two main phenotypes of macrophages: M1 and M2, each with distinct functions and characteristics. M1 macrophages have pro-inflammatory characteristics, and are responsible for antigen presentation and protection against tissue pathogens. They are primarily involved in anti-tumor immunity. Macrophages exhibit unique gene expression patterns and transcription factors depending on their tissue of origin. Based on their location and function, they can be broadly classified into different categories as well [12]. Moreover, fully differentiated macrophages transplanted from one site to another can adapt their gene expression profiles to the new environment [13]. Conversely, M2 macrophages, often called anti-inflammatory macrophages, exhibit a role of homeostatic balancing in the tissues [12]. Tumors alter M2 subsets of macrophages, known as tumor-associated macrophages, to enhance their own protection and growth. TAMs can exhibit diverse phenotypes, typically polarized towards M2-like characteristics in the TME, supporting tissue repair, tumor growth, and immune suppression. In the context of HCC, TAMs also have an M2-like phenotype, with anti-inflammatory properties facilitating tumor growth [5].

**Figure 1:**
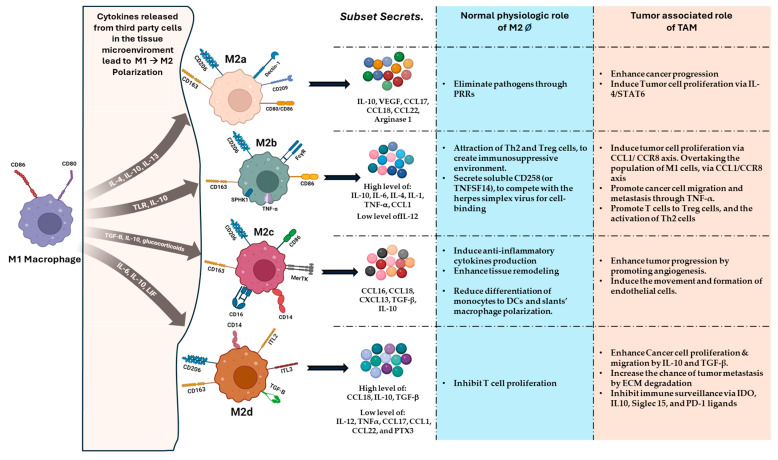
M2 macrophages in terms of subsets, immunophenotyping, polarization cytokines, secreted cytokines, normal physiologic role, and pathologic role in cancer.

M2-like macrophages can be further classified into four subsets, specifically M2a, M2b, M2c, and M2d (Figure 1). Although the four subsets display distinct phenotypic markers, gene expression profiles, functional activities, and distinct cytokines, they are all characterized by high levels of IL-10 and low levels of IL-12 [14,15].

M2a macrophages are a widely studied M2 subset, polarized under stimulation with IL-4, IL-10, and IL-13. They are characterized by high expression of CD206, CD209, and Dectin-1, as well as moderate to low expression of CD14, CD163, and CD80/86. M2a cells produce cytokines such as IL-10, chemokines such as CCL17, CCL18, and CCL22, and the amino-acid catabolizing enzyme Arg1 [16]. M2a has the capacity to sense and eliminate invading pathogens, such as bacteria, parasites, and fungi, through pattern recognition receptors (PRRs) [17]. M2a macrophages express CD80^+^/CD86^+^, which renders M2a macrophages poor inducers of T cell activation and proliferation. As a reaction to tissue damage, the release of IL-4 triggers the differentiation of macrophages into M2a, facilitating tissue remodeling via fibronectin secretion [14]. Fibronectin enhances tumor growth by stimulating the proliferation, infiltration, and motility of tumor cells. Considering its detrimental role in cancers, fibronectin is now known as a potential target for cancer therapy [18]. The pro-tumoral effects of M2a macrophages include the synergistic secretion of vascular endothelial growth factor (VEGF) and CCL18, leading to angiogenesis and increased migration of cancer cells. In several types of solid tumors, the pathway signaling of IL-4/STAT6 pathway is used by M2a cells to promote cancer progression and stimulate the proliferation of tumor cells [19]. Both tumor cells and M2a release IL-4, which promotes the polarization of additional macrophages towards M2a and leads to the production of more IL-4, thereby creating a positive feedback loop. M2a macrophages promote tumor progression through tumor-released factors and IL-4/IL-13 signaling [20].

Introducing TLR agonists (such as LPS) and immune complexes (ICs) to M1 macrophages leads to the downregulation of IL-12 and the upregulation of IL-10, which is then converted into the M2b subset, also known as regulatory macrophages [21]. M2b macrophages have specific characteristics, such as the Fcγ receptor (FcγR) crosslinking, elevated IL-10 secretion, and reduced IL-12 levels. Secreted IL-4 from M2b macrophages influences Th1-cell responses in favor of Th2-cell responses [22]. A distinguishing feature of M2b cells is their ability to generate significant amounts of CCL1, which plays a vital role in upholding their unique characteristics. The high levels of CCL1 produced by M2b macrophages attract Th2 and Treg cells, creating an immunosuppressive environment. CCL1 binding to CCR8 on tumor cells drives their proliferation, migration, and metastasis [23,24]. Notably, when CCL1 is suppressed, M2b cells undergo a shift towards M0 or M1 macrophages [24]. A distinguishing characteristic of M2b is LIGHT (known as CD258 or TNFSF14), a secreted protein that competes with the cell biniding for the herpes simplex virus [14]. Sphingosine kinase 1 (SPHK1) displayed exclusive upregulation in M2b cells. The coexpression of CD86 and TNF-α can distinguish M2b from the other macrophage subsets. However, CD86 was also expressed in the M2b subset. It is noteworthy that M2b macrophages were identified as TAMs in patients with intermediate-stage hepatocellular carcinoma (HCC) [24]. Few M2b macrophages can be identified in early stage HCC; they increase in preponderance in parallel with increasing tumor stages, gradually overtaking the population of M1 cells, with M2b cells and the CCL1/CCR8 axis being linked [5]. In both HCC and breast cancer, M2b macrophages promote cancer cell migration and metastasis through TNF-α. The interaction between infiltrating B cells and TME leads to the production of M2b cells and increased levels of IL-10 and CCL1 [25]. The secretion of IgG by infiltrating B cells results in the formation of immune complexes with tumor antigens. Subsequently, these complexes engage with Fcγ receptors located on the macrophages in the TME [26]. In the same way, the involvement of the TLR4 signaling pathway in the polarization of M2b macrophages was identified, and blocking it prevented the upregulation of M2b markers. M2b macrophages secret cytokines such as IL-10, IL-6, and IL-1, promoting the transformation of T cells from naïve T cells into Treg cells and activating Th2 cells, which suppress the function of cytotoxic T-cells [27].

M2c macrophages are also a subtype of M2 macrophages that are activated by TGF-β, IL-10, or glucocorticoids. M2c cells show high expression of CD163, Mer-tyrosine kinase (MerTK), and Tie2 on their cell surface, alongside moderate levels of CD86 and CD206, in addition to the monocyte basic markers CD14 and CD16 [16]. M2c macrophages are characterized by their production of CCL16, CCL18, and CXCL13, as well as TGF-β and IL-10. M2c macrophages are similar to M2a in L-arginine and Arg1 metabolism [28]. The secretion of IL-10 by M2c cells delays the differentiation of monocytes to dendritic cells (DCs) and slows macrophage polarization. In response to IL-10 exposure, macrophages progressively upregulate the expression of genes linked to anti-inflammatory activities [29]. M2c macrophages are alternatively referred to as “acquired deactivation macrophages” due to the loss of their ability to repolarize back to M1. Blocking STAT3-mediated signaling can potentially convert M2c macrophages back to M1 [30]. M2c macrophages play an immunoregulatory role by suppressing M1-favored inflammation through various mechanisms [17], including the interruption of M1 polarization and release of pro-inflammatory cytokines and chemokines, such as CCR2 and CCR5. M2c macrophages are known for their ability to produce a high amount of anti-inflammatory cytokines, leading to tissue remodeling by the degradation of the extracellular matrix (ECM) [31]. Growing evidence suggests that M2c macrophages are involved in supporting tumor progression by promoting angiogenesis and assisting in the formation and movement of endothelial cells, both in vitro and in vivo. Disease severity is correlated with the number of circulating M2c macrophages [14,32].

M2d macrophages are abundant in ascites, showing a correlation with high levels of IL-6 and leukemia-inhibitory factor (LIF). The distinctive features of polarized M2d macrophages include a specific phenotype and cytokine production pattern. This includes high levels of IL-10, low levels of IL-12, and increased expression of CD14, CD163, TGF-β, Ig-like transcript 2 (ILT2), and Ig-like transcript 3 (ILT3). These characteristics provide compelling evidence of their strong capability to inhibit T cell proliferation [33]. M2d macrophages are distinguished by their ability to produce significant amounts of CC18, while showing decreased levels of TNFα, CCL17, CCL1, CCL22, and PTX3 [16]. IL-6 and LIF play a role in enhancing the self-consumption of M-CSF by macrophages during M2d polarization. The presence of IL-6 and LIF not only boosts M-CSF uptake, but also plays a crucial role in the complete differentiation of macrophages into the M2d phenotype. Furthermore, LIF prompts the secretion of IL-6 in macrophages, boosting their capacity to capture M-CSF [33]. M2d macrophages promote tumor progression via two primary mechanisms: firstly, by producing IL-10 and TGF-β, which induce the proliferation and migration of cancer cells, and secondly, by contributing to angiogenesis and the degradation of the extracellular matrix, which facilitate metastasis. M2d cells release IL-6, a cytokine that has been associated with the development of different tumor types, including hepatocellular cancer [34]. Through the IL-6/JAK/STAT3 canonical pathway, the expression of genes involved in anti-apoptosis, proliferation, angiogenesis, metastasis, and drug resistance is upregulated. M2d can suppress normal immune responses, enabling tumor cells to evade immune surveillance through the expression of IL-10, IDO, PD-1 ligands, and Siglec 15 ligands. In brief, the common characteristics of the distinct M2 macrophage subsets include their contribution to tumor development and their ability to hinder the effectiveness of adaptive immune responses [14,35]. Research on the immunosuppressive TAMs, particularly regarding which M2 macrophages subset exists within the TME depending on histology, is primitive. TAM subsets can assume an M2-like phenotype. They can induce efficient immune suppression synergistically in a synchronized or independent manner. While studies have explored the diverse functions, markers, and cytokine interactions of M2 macrophage plasticity [12,16], a lack of clinical evidence prevents the consistent identification of distinct M2 macrophage subsets based on these markers.

### 2.2. Immune Response Modulation by TAMs

The polarization of macrophages into M2 TAMs is triggered by the release of IL-10 and lipid sphingosine 1-phosphate (S1P) signaling from necrotic tumor cells [36]. When TAMs undergo transformation into the M2-like phenotype, there is a decrease in the secretion of inducible nitric oxide synthase (iNOS) and nitric oxide (NO). TAMs also induce matrix metalloproteinase (MMP) expression, resulting in the cleavage of the Fas ligand from cancer cells [37]. The role of the Fas ligand, or CD95L, has been widely acknowledged as a receptor–ligand system that triggers apoptosis, ensuring immune homeostasis and playing a vital part in eliminating cancer cells [38].

TAMs play a critical role in modulating both local and systemic immune responses [39]. Locally, TAMs contribute by secreting suppressive pro-tumor cytokines and growth factors that can inhibit anti-tumor immunity, promote angiogenesis, and facilitate tumor invasion [40]. In the initial phase of tumor development, classically activated macrophages generate nitric oxide (NO), resulting in significant tumor cell death. However, as tumors grow, the release of prostaglandins-E2 (PGE2) and IL-10 by cancer cells hinders the immune responses mediated by M1 proinflammatory cytokines [23]. In addition to making the cancer cells resistant to chemotherapy, the pro-tumor effect of immunosuppressive macrophages hinders the ability of NK cells and T cells to eliminate it [37]. In vitro studies show that M2-like TAMs lack tumor-associated antigens and do not aid in the priming of T cells and NK cells against cancer cells. An association has been identified between the phenotype of macrophages and IL-4 and TGF-β [39,41]. The latter can suppress the secretion of IL-12 via macrophages, hindering the proliferation of NK cells and cytotoxic T cells and inducing their exhaustion through arginase 1 (ARG1), indoleamine 2,3-dioxygenase (IDO), IL-10, programmed death ligand 1 (PD-L1), and TGF-β [11,39,42]. TAMs can systemically influence the activity of T cells and dendritic cells by secreting anti-inflammatory cytokines that can impact distant organs [43]. TAMs can produce mediators to modulate TME to support tumor proliferation and protect against apoptosis through the secretion of growth factors such as the tumor necrosis factor (TNF)-α, interleukin (IL)-1β, IL-6, C-C motif chemokine (CCL)2, and others [42].

## 3. Local Tumor Microenvironment

### 3.1. TAMs and Immunosuppressive Cytokine Production

TAMs in the TME often exhibit anti-inflammatory M2 macrophages, characterized by the production and secretion of immunosuppressive cytokines such as TGF-β, IL-10, IL-4, IL-13, IL-1RA, and macrophage colony-stimulating factor (M-CSF). Moreover, M2-like TAMs demonstrate lower expression of inflammatory cytokines such as IL-6, IL-12, IL-23, and TNF-α. M2-like macrophage phenotypes are CD163 high, CD206 high, CD200R high, CD209 high, and CD301 high, and they express chemokines ligands such as CCL1, CCL17, CCL18, CCL22, and CCL24. M2-like macrophages support cell proliferation and play a role in promoting angiogenesis through platelet-derived growth factor (PDGF) and insulin-like growth factor (IGF). These cytokines create an environment that inhibits the function of cytotoxic T lymphocytes (CTLs), blocks Th1-type immune activity, promotes anti-immunity Th2-type activity, and suppresses the cytotoxic activity of natural killer (NK) cells, thus allowing tumors to evade immune surveillance and thereby promoting tumor cell growth, drug resistance, and angiogenesis [20,42,44,45,46].

### 3.2. TAMs Related to Angiogenesis and Tumor Progression

TAMs, known for their angiogenic properties, actively release VEGF, a growth factor that stimulates the formation of blood vessels, ensuring a steady flow of nutrients and oxygen to the tumors. Angiogenic support boosts tumor growth and weakens the immune system [47]. The pro-angiogenic factors secreted by TAMs are VEGF, epidermal growth factor (EGF), placental-derived growth factor (PlGF), platelet-derived growth factor (PDGF), TGF-β, TNF-α, IL-1β, IL-8, CCL2, CXCL8, and CXCL12 [48]. The presence of Neuropilin-1 (Nrp1) is essential for guiding TAMs into hypoxic niches in response to Semaphorin 3A (Sema3A). The removal of Nrp1 has been shown to boost the immune system’s response against tumors by impeding the growth of new blood vessels. After infiltrating the hypoxic niches, macrophages downregulate the expression of Nrp1 in TAMs, causing TAMs to become trapped at the site [49]. The occurrence of TAMs in tumors is directly linked to elevated levels of VEGF and microvascular density. Macrophage-produced Wnt Family Member 7B (WNT7b) is crucial for tumor progression by enhancing VEGF-A expression in endothelial cells. TAMs produce VEGF-A through HIF-1α expression, which induces tumor angiogenesis. Hypoxic TME polarizes recruited macrophages into the M2 phenotype and TAMs induce tumor angiogenesis [50]. In addition, TAM-induced MMPs also contribute to tumor angiogenesis. TAMs release thymidine phosphorylase (TP) and urokinase-type plasminogen activator (uPA), promoting tumor angiogenesis with increased vascular invasion and ECM degradation [51]. TAMs stimulate endothelial cells (ECs) and release YKL-40, a secreted glycoprotein known as chitinase-3-like-1. YKL-40 activates the MAPK signaling pathway in ECs, leading to increased production of VEGFR-1 and VEGFR-2, ultimately facilitating vessel formation [52].

### 3.3. TAMs and Extracellular Matrix Remodeling

The presence of TAMs is crucial for ECM remodeling. TAMs degrade ECM, stimulate fibroblasts, and synthesize various proteins. This leads to the formation of a reactive stroma, thereby promoting tumor cell invasion, proliferation, and angiogenesis [53]. The regulation of TAMs in the TME is intricate. Cancer cells secrete M-CSF along with various chemokines and proteins. These factors modulate TAM function in the TME. During collagen fibrillogenesis, TAMs may adjust their function depending on factors produced by cancer-associated fibroblasts (CAFs). Both TAMs and CAFs collaborate in depositing, cross-linking, and aligning fibrillar collagens, thus shaping the tumor’s extracellular matrix, which ultimately affects the TME and the immune response [54]. TAMs’ specialized function is highlighted by their secretion of MMP11, which is instrumental in promoting the migration of cancer cells. The CCL2–CCR2 signaling pathway mediates this mechanism, which underscores the precise and selective actions of TAMs across various cancer subtypes. Elevated levels of B7-H3 expression in TAMs contribute to the activation of regulators, such as MMP2, VEGF-A, and TGF-β, involved in ECM construction and angiogenesis, which contribute to the metastatic spread of tumors [11,55]. Podoplanin-expressing macrophages (PoEMs), a different subset of TAMs, facilitate lymphangiogenesis and lymphoinvasion and aid in the progression of cancer. The selective adherence of PoEMs to the walls of lymphatic vessels highlights the precise and specialized interactions of TAMs in the TME. For example, TAMs in gastric cancer have been found to express DAB2, which participates in haptotaxis, integrin recycling, and transduction. The versatile functions of TAMs in ECM remodeling make them crucial for the progression and spread of cancer [56,57].

### 3.4. TAMs’ Impact on the Development of HCC

The development of HCC relies on a tumor microenvironment that enhances tumor cell growth and suppresses the immune cells’ ability to kill tumor cells. TAMs express triggering receptor expressed on myeloid cells-1 (TREM-1) and upregulate C-C motif ligand (CCL)-20 via the ERK/nuclear factor kappa B (NF-κB) pathway. They also recruit Tregs and the suppressive immune cells that cause CD8+T cell apoptosis and dysfunction. TAMs express Siglec-10 and myristoylated alanine-rich C-kinase substrate (MARCKS), leading to M2 macrophage polarization, immunosuppression, and immune escape, and ultimately to a poor prognosis. Cancer cells have specific nutrient requirements to fuel their growth and rapid division. By enhancing MMP-9 expression, TAMs are associated with elevated VEGF-A levels, inducing tumor growth and tumor angiogenesis in HCC, mediated by the type 1 insulin-like growth factor (IGF-1) signaling pathway through the PI3-K and MAPK pathways [58,59,60]. A clinical trial treating colorectal liver metastasis via radiofrequency ablation assessed the treatment’s efficacy by measuring the lymphocyte to monocytes/macrophages ratio (LMR). Low LMR levels were associated with a weakened immune response that promoted the TME for tumor growth, mirrored in the 34 months of overall survival of these patients, while high LMR levels had favorable outcomes in terms of tumor burden reduction and improved overall survival to 55 months [61]. Another trial of treating recurrent HCC patients with percutaneous radiofrequency ablation showed that post-recurrence survival of patients who relapsed and were treated within the same tumor lesion was higher than those with generated distant recurrence or those with BCLB C recurrence (averages: 39 months vs. 27 months vs. 7 months, respectively) [62], suggesting the substaintial role of TME in HCC.

TAMs produce cytokines that induce epithelial-mesenchymal transition (EMT) and cancer stem cell (CSC) characteristics in HCC cells, thus supporting invasiveness and metastasis through the secretion of TGF-β1 and TNF-α. Cancer cells undergo EMT through IL-6 secretion to transform into stem-like cells with self-renewal capabilities in hypoxic conditions [63]. Within the HCC tumor microenvironment, the presence of CAFs significantly impacts the behavior of TAMs. In addition to prompting TAMs to adopt the M2 phenotype, CAFs also stimulate the production of CXCL12 [64], which prompts M2 cells to secrete plasminogen activator inhibitor-1 (PAI-1), contributing to the promotion of EMT and enhancing the malignant features of HCC cells. The TAM-mediated angiogenesis described above is important in tumor development, as well as in tumor invasion and metastasis [3,65].

## 4. Therapeutic Strategies for Suppressing TAMs in HCC and Other Cancers

### 4.1. Monoclonal Antibodies

Monoclonal antibodies targeting TAM-related pathways have shown promise in preclinical studies. Notable targets are CSF-1R (colony-stimulating factor 1 receptor), CD163, and SIRPα (CD172a), which are critical for TAM survival and function [66,67]. Using a CSF-1R blocker (Pexidartinib), in the form of hydrogel loaded with Pexidartinib (PLX)-encapsulated nanoparticles, facilitates the local and systemic delivery of anti-programmed cell death protein 1 (anti-PD-1) antibody-conjugated platelets and inhibits tumor recurrence after surgery [68]. Antibodies targeting or blocking TAMs can prevent their recruitment and survival, inhibiting TAMs’ function [69], and induce reprogramming, proliferation, and activation of CD8+ T cells [70] (Table 1).

OR2805, an anti-CD163 monoclonal antibody, a marker specific to M2 macrophages, is another target. By targeting CD163 or triggering receptor expressed on myeloid cells-1 (TREM-1) (known as PY159) in TAMs with monoclonal antibodies, TAMs in the TME of HCC can be eliminated or reprogrammed, potentially reversing the immunosuppressive environment of HCC’s TME [59,71]. CD47, expressed in cancer cells, binds macrophage SIRPα to inhibit the cytoskeletal rearrangements necessary for phagocytosis. The first-in-class monoclonal CD47-targeting antibody magrolimab has been used in several clinical trials (Table 1). Unfortunately, the trials were terminated because of its poor efficacy and systemic toxicities [72,73].

**Table 1:** Clinical trials for TAM blocking, inhibitors, or depletion.

Category	Drug or Substance	2nd Combination Therapy	Tumor Type	Trial Design	Clinical Trials
CSF-1R Inhibitors	PLX3397	Monotherapy	Mucosal Melanoma	Phase II	NCT02071940
	Pexidartinib	Monotherapy	Advanced solid tumors	Phase I	NCT02734433
	Pexidartinib	Monotherapy	Pigmented Villonodular Synovitis (PVNS) or (GCT-TS)	Phase III	NCT02371369
	Pexidartinib, PLX3397	Monotherapy	Refractory Leukemia, sarcoma, or neurofibroma	Phase I/II	NCT02390752
	PLX3397	Monotherapy	Relapsed Acute myeloid leukemia	Phase I/II	NCT01349049
	PLX7486 (Plexxikon)	Monotherapy	Advanced-stage or metastatic solid tumors	Phase I	NCT01804530
	Vimseltinib (DCC-3014)			Phase I/II	NCT03069469
	ARRY-382			Phase I	NCT01316822
	LY3022855 mAb (IMC-CS4)	Monotherapy	Breast and prostate cancers	Phase I	NCT02265536
		Monotherapy	Advanced Solid Tumors	Phase I	NCT01346358
	AMG820 mAb	Monotherapy	Advanced Solid Tumors	Phase I	NCT01444404
	Emactuzumab (RG715)	Anti-CD40L selicrelumab (RO7009789)	Advanced Solid Tumors	Phase I	NCT02760797
		Paclitaxel + anti-VEGFA mAb bevacizumab	Primary peritoneal cancer	Phase II	NCT02923739
		Monotherapy and with paclitaxel	Solid tumors	Phase I	NCT01494688
	Cabiralizumab (FPA008)	Vemurafenib, Cobimetinib	Melanoma	Phase I/II	NCT03101254
	PLX3397 (Pexidarnitib)	Pembrolizumab	Solid tumors, Melanoma	Phase I/II	NCT02452424
		Durvalumab	Metastatic/Advanced Pancreatic or Colorectal Cancers	Phase I	NCT02777710
	LY3022855 mAb (IMC-CS4)	Pembrolizumab	Pancreatic adenocarcinoma cancer	Phase I	NCT03153410
		Durvalumab or Tremelimumab	Advanced solid tumors	Phase I	NCT02718911
	RO5509554 (Emactuzumab)	MPDL3280A (Atezolizumab)	Advanced Solid tumors	Phase I	NCT02323191
	AMG820 mAb	Pembrolizumab	Solid tumors	Phase I/II	NCT02713529
	BLZ945	PRD001	Advanced solid tumors	Phase I/II	NCT02829723
	Cabiralizumab	Nivolumab	Advanced solid tumors	Phase I	NCT02526017
	PLX3397 (Pexidarnitib)	Paclitaxel	Advanced solid tumors	Phase I	NCT01525602
	I-SPY2	Standard Chemotherapy (Trastuzumab)	Breast cancer	Phase II	NCT01042379
	PD-0360324 mAb	Cyclophosphamide	Ovarian cancer	Phase II	NCT02948101
	PLX3397 (Pexidarnitib)	Sirolimus (Rapamycin)	Sarcoma	Phase I/II	NCT02584647
		Eribulin	Metastatic breast cancer		NCT01596751
	PLX3397 (Pexidarnitib)	Radiotherapy + Antihormone therapy	Prostate cancer	Phase I	NCT02472275
		Radiotherapy + Temozolomide	Glioblastoma	Phase I/II	NCT01790503
	ARRY-382	Pembrolizumab	Advanced solid tumors	Phase I/II	NCT02880371
	JNJ-40346527	Daratumumab (CD38)	Advanced prostate cancer	Phase I	NCT03177460
	JNJ-40346527	Monotherapy	Healthy volunteer	Phase I	NCT01054014
	PF-04136309	FOLFIRINOX	Advanced Pancreatic cancer	Phase I	NCT01413022
CCR2 Inhibitors	PF-04136309	Gem/Nab-P	Metastatic pancreatic cancer	Phase II	NCT02732938
	MLN1202	Monotherapy	Bone metastasis	Phase II	NCT01015560
	BMS-813160 (CCR2/CCR5 antagonist)	Nivolumab	Advanced solid tumors	Phase I/II	NCT03184870
	CNTO 888 (Carlumab)	Gemcitabine/paclitaxel Carboplatin/doxorubicin	Advanced solid tumors	Phase I	NCT01204996
	PF-04136309	FOLFIRINOX	Advanced Pancreatic tumors	Phase I	NCT01413022
	BMS-813160	Nivolumab, Ipilimumab, Relatlimab	Advanced renal cell carcinoma	Phase II	NCT02996110
CD40 blockers	*SEA-CD40*	pembrolizumab, carboplatin, and pemetrexed.	Advanced Cancers	Phase II	NCT04993677
	APX005M	Nivolumab and Ipilimumab	Advanced melanoma or renal cell carcinoma (RCC)	Phase I	NCT04495257
	Selicrelumab (RO7009789)	Atezolizumab	Metastatic solid tumors	Phase I	NCT02304393
	CP-870,893	Pembrolizumab	Solid Tumors	Phase I	NCT02225002
	Selicrelumab (RO7009789)	Vanucizumab or Bevacizumab	Advanced solid tumors	Phase I	NCT02665416
	NG-350A	Pembrolizumab	Advanced Epithelial Tumours	Phase I	NCT05165433
	CDX-1140	pembrolizumab or chemotherapy	Advanced Malignancies	Phase I	NCT03329950
SIRP-a Blockers	IBI188	Monotherapy	advanced malignant tumors	Phase I	NCT03717103
	Magrolimab (Hu5F9-G4)	Cetuximab	Solid Tumors and Advanced Colorectal Cancer	Phase I/II	NCT02953782
	*ALX148*	Pembrolizumab	Head and neck squamous cell carcinoma (HNSCC)	Phase II	NCT04675294
	PF-07901800 (TTI-621)	Rituximab, Nivolumab	Hematological malignancies, Solid Tumors	Phase I	NCT02663518
	*AK117*	Monotherapy	advanced solid tumors	Phase I	NCT04728334
		AK104	Solid tumors		NCT04349969
	NI-1801	Pembrolizumab, paclitaxel	Mesothelin Expressing Solid Cancers	Phase I	NCT05403554
	BI 765063	BI 754091	Advanced solid tumor	Phase I	NCT03990233
Others	Imalumab (MIF)	Monotherapy	Solid tumors	Phase I	NCT01765790
	FP-1305 / bexmarilimab	Monotherapy	Cancers	Phase I/II	NCT03733990
	BDC-1001 (TLR7/8)	Alone or Nivolumab	HER2^+^ solid tumors	Phase I/II	NCT04278144
	CNTO 888 / Carlumab (CCL2)	Monotherapy	Metastatic Castrate-Resistant Prostate Cancer	Phase II	NCT00992186
	Vanucizumab (RG7221) (VEGF/ Ang2)	alone or with Atezolizumab	Advanced or Metastatic Solid Tumors	Phase I	NCT01688206
	PY314 (TREM2)	PY314 + Pembrolizumab	Advanced Solid Tumors	Phase I	NCT04691375
	JTX8064 (LILRB2)	Alone or with pimivalimab	Advanced refractory solid tumors	Phase I/II	NCT04669899
	Epacadostat (IDO)	Monotherapy	Ovarian cancer genitourinary tumors	Phase II	NCT01685255
	Eganelisib (IPI-549) (Tyrosine kinase Inhibitor) (PI3Kγ)	Monotherapy or with Nivolumab	Advanced solid tumors	Phase I	NCT02637531
	CT-0508 (CAR-Macrophage)	Pembrolizumab	HER2^+^ solid tumors	Phase I	NCT04660929
	CCR5 antagonist (CCR5)	PD-1	Refractory Microsatellite Stable CRC	Phase I	NCT03274804
	BO-112 (TLR3)	Pembrolizumab	Colorectal or Gastric/GEJ Cancer With Liver Metastasis	Phase II	NCT04508140
	SHR2150 (TLR7)	Anti-PD-1 and Anti-CD47	Metastatic solid tumors	Phase I/II	NCT04588324
	TransCon (TLR7/8)	Pembrolizumab	Advanced or Metastatic Solid Tumors	Phase I/II	NCT04799054
	CMP-001 (TLR9)	Pembrolizumab	Advanced Melanoma	Phase I	NCT03084640
	GEN-1 (IP IMNN-001) (IL-12)	Monotherapy	Neoadjuvant Chemo in Ovarian Cancer	Phase I	NCT02480374

### 4.2. Small Molecule Inhibitors

**Figure 2:**
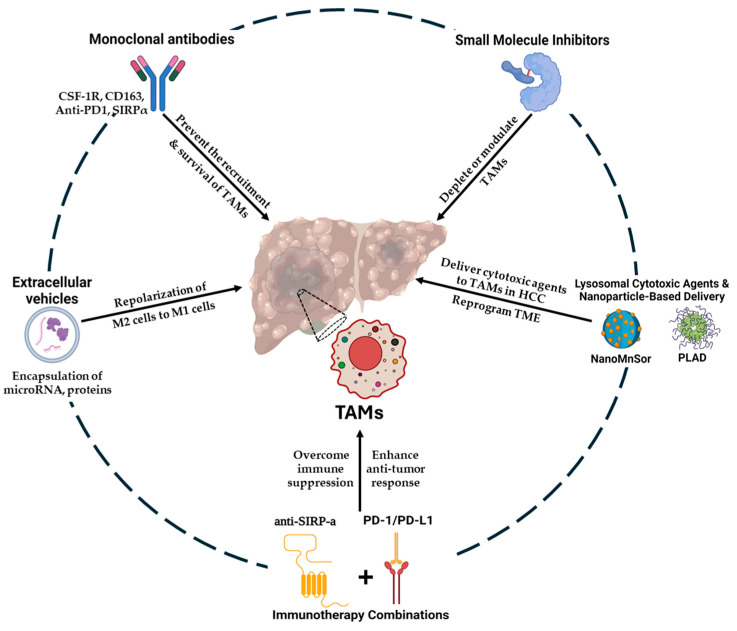
Tumor-associated macrophages therapeutic strategies for HCC, illustrated in the figure.

Small molecule inhibitors targeting TAMs are emerging as a strategic approach in the treatment of hepatocellular carcinoma (HCC), focusing on key pathways such as CCR2 and p38 MAPK to deplete or modulate TAMs. CCR2, a chemokine receptor 2 which is involved in the recruitment of monocytes to the TME, can be effectively inhibited by small molecules. The goal is to reduce TAM infiltration and decrease their role in tumor progression, as well as enhance systemic immune responses [74]. Treating mice with 747, a CCR2 antagonist, was found to double the infiltration of CD8^+^ T cells in liver cancer by inhibiting TAM suppression. Moreover, 747 led to a 43% reduction in TAMs and an inhibition of tumor growth via CD8+ T cell-dependent pathway. The 747 exhibited little toxicity, as indicated by the mice’s weight, serum, liver, and kidney enzymes levels [75]. The p38 MAPK (mitogen-activated protein kinase) pathway, a signaling molecule involved in macrophage activation and inflammatory responses, represents another critical target. The p38 MAPK pathway is critical for TAM activation and survival. Inhibitors of p38 MAPK can disrupt the inflammatory signaling that supports TAMs’ survival and function. By targeting these pathways, small molecule inhibitors not only aim to reduce the number of TAMs but also to alter their phenotype from pro-tumorigenic to anti-tumorigenic. This can be used to enhance the efficacy of conventional therapies and potentially improve patient outcomes in HCC [17,76]. Lenvatinib is a multiple tyrosine kinase receptor inhibitor that ultimately inhibits p38 MAPK. Lenvatinib initiates its activity via the inactivation of the p38 MAPK/NF-κB pathway, resulting in decreased levels of circulating TNF-α and IL-6, reducing cancer cell migration and invasion, and enhancing M1 polarization of TAMs in a mouse HCC model [77,78,79] (Figure 2 & Table 2).

### 4.3. Lysosomal Cytotoxic Agents and Nanoparticle-Based Delivery

Several targeted delivery systems, including nanoparticles and liposomal formulations, were adopted to deliver cytotoxic agents specifically to TAMs in HCC. Furthermore, the use of nanoparticles can provide controlled release of and prolonged exposure to cytotoxic agents, increasing the likelihood of achieving therapeutic outcomes. By leveraging the specificity and targeted delivery capabilities of nanoparticles, this approach holds promise for more effectively managing TAMs and improving treatment responses in HCC [80] (Figure 2 & Table 2).

Liposomal delivery modifies the pharmacokinetics of doxorubicin, resulting in a profound alteration of the drug’s immune modulatory activity. Encapsulating doxorubicin in liposomes targeted to TAMs can enhance the drug’s specificity and reduce off-target effects. The pegylated liposomal doxorubicin (PLAD) nanotheranostic platform not only enhances immune changes but also inhibits pro-tumoral macrophages in TME, potentially synergizing with its cytotoxic chemotherapy effects [81]. Another approach was used for liposomal localized delivery of sorafenib or regorafenib in a rabbit tumor model. The mechanism of using drug-eluting embolic transarterial chemoembolization (DEE-TACE) is found to improve overall survival of HCC. The optimization of TACE by local delivery of sorafenib or regorafenib avoids systemic release, thus preventing systemic side effects while increasing local effects and efficacy [82]. Although liposomal delivery has shown promising results, further studies are needed to study its effects on TAMs.

Another trial for the synthesis and evaluation of nano-sorafenib-manganese oxide (NanoMnSor), a nanoparticle drug carrier that efficiently co-delivers oxygen-generating MnO_2_ and sorafenib into HCC, has shown promise in clinical trials. Using ultrasound, the macrophages’ MnO_2_ microbubbles resonated, indicating their heightened activity. The NanoMnSor treatment effectively reduces tumor vascularization, overcoming hypoxia-driven resistance to sorafenib and successfully inhibiting the growth of primary tumors and distant metastasis in a mouse orthotopic HCC model, leading to enhanced overall survival. Moreover, NanoMnSor has the ability to reprogram the immunosuppressive tumor microenvironment (TME) by reducing the infiltration of TAMs secondary to hypoxia, stimulating the polarization of macrophages towards the immunostimulatory M1 phenotype, and increasing the presence of CD8+ cytotoxic T cells within tumors. Consequently, it significantly improves the efficacy of anti-PD-1 antibody treatment and whole-cell cancer vaccine immunotherapies [83].

### 4.4. Immunotherapy Combinations

Combining immunotherapy with checkpoint inhibitors (e.g., anti-PD-1/PD-L1, anti-SIRP-α/CD47, CD40/CD40L, or anti-Siglec-10/CD24) or systemic therapies with TAM depletion aims to enhance the anti-tumor response against HCC by creating a favorable immune environment [84,85] (Figure 2 & Table 2).

### 4.5. Extracellular Vehicles Therapy

The release of extracellular vehicles (EVs) or exosomes by all cell types facilitates the transmission of information and communication between individual cells. The EVs released by TAMs during antitumor therapy have been discovered to have advantages and disadvantages [86]. The encapsulation of microRNA (miR) in EVs makes it a vital mechanism of gene expression. TAM-derived EVs that carry miR-146a contribute to the repolarization of M2 cells to M1 cells, whereas miR-19a-3p and miR-155 contribute to the polarization of M1 cells to M2 cells [39] (Figure 2 & Table 2).

**Table 2:** Tumor-associated macrophages therapeutic strategies for HCC, illustrated in the summary table.

Anti-TAMs Molecule	Mechanism	Drugs Names	References
**MoAb** (CSF-1R, CD163, Anti-PD-1, SIRP-α)	Block and prevent TAM recruitment, decrease TAM survival	**CSF-1R** (BLZ945, Imatinib, PLX5622, GW2580), **CD163** (M130), **Anti-PD-1** (Pembrolizumab, Nivolumab, Cemiplimab, **SIRP-a** (BI735063, CC95251, DS-1103, LM-101, BR1051)	[61,62,63,64,65,66,67,68,87,88,89]
**Extracellular vehicles**	Repolarize M2 to M1 macrophages	Exosomes Encapsulation with different molecules, such as targeted microRNA or specific proteins	[81,82,83,84]
**combination** (PD-1/PD-L1, SIRP-α)	Overcome immune suppression in TME, enhance anti-tumor response	Combination of MoAbs.	[79,80]
**Lysosomal cytotoxic agents and Nanoparticle-based delivery**	Cytotoxicity towards TAMs	Nano-Manganese sorafenib (NanoMnSor), Pegylated liposomal doxorubicin (PLAD)	[75,76,77,78]
**Small molecule inhibitors**	TAM blocking or depletion	CCR2, Lenvatinib	[69,70,71,72,73,74]

TAM-derived EVs are associated with immunosuppressive microenvironments. Both miR-29a-3p and miR-21–5P inhibit STAT3 in CD4+ T cells, leading to a Treg/Th17 cell imbalance and facilitating cancer progression. The miR-223 is transferred to cancer cells by TAM-derived EVs [90]. The transfer of miR-21–5p and miR-155–5p in colorectal cancer cells enhances metastasis. The activation of the PI3K-Akt signaling pathway is facilitated by a specific protein called Apolipoprotein E (ApoE), which is present in M2 TAM-derived exosomes [91]. The presence of this protein stimulates the migration of gastric cancer cells. Another hypothesis is that M2 TAM-derived EVs exhibit molecular profiles akin to M1 TAMs, suggesting their ability to activate the immune response. Notably, this finding is further supported by the presence of STING and proteins associated with TLR signaling. Hence, there is a need for further exploration of the effects of TAM-derived EVs [92].

## 5. Challenges and Future Directions

### 5.1. TAMs Heterogeneity and Dynamic Plasticity

TAMs are not a homogeneous population; they exhibit considerable variability in their functions and markers. Understanding this diversity and plasticity is crucial for developing effective therapies. When the TME undergoes changes, TAMs can adapt by altering their phenotype, impacting the HCC TME [93]. The sub-localization of macrophages within the TME plays a role in determining the subsets and functions of macrophages. Based on the spatial transcriptomics profiling of TAMs, they are classified into immunostimulatory macrophages, inflammatory macrophages (Inf-TAMs), immunoregulatory macrophages (Reg-TAMs), angiogenic macrophages (Ang-TAMs), interferon-mediated regulatory macrophages, and a CD169^+^ (*SIGLEC-1)* macrophage [94]. Investigating how TAM populations change in response to treatment and tumor progression will inform adaptive therapeutic strategies [95].

Within the complex TME, TAMs are recruited by vascular endothelial and perivascular cells, resulting in their polarization. This process produces angiogenic factors that promote blood vessel growth, thereby aiding tumor development. The interactions between immune cells (T cells, B cells, NK cells, and MDSCs) and macrophages hinder T cells’ infiltration into tumors and promote phenotypic conversion of macrophages to the M2 phenotype, thus further fueling tumor development and immune suppression [96]. The recruitment of CAFs and TAMs plays a crucial role in the extracellular matrix remodeling and tumor invasion facilitated by CAFs [97,98].

### 5.2. Complex Interactions and Resistance Mechanisms

TAMs can contribute to resistance to different treatments, including chemotherapy, targeted therapies, immunotherapies, and radiation therapy [44,99,100]. They can promote immune evasion, create a pro-tumorigenic environment, and interfere with drug efficacy [11]. HCC cancer may develop resistance to TAM-targeting therapies, including compensatory pathways by the upregulation of alternative immune suppressive pathways or the recruitment of other immune cell types that can support tumor growth [101]. Standout pathways for the recruitment and maturation of TAMs include the CSF1/CSF1R and CCL2/CCR2 axes. Macrophage survival and maturation heavily relies on the CSF1/CSF1R pathway, whereas the CCL2/CCR2 axis primarily regulates monocyte trafficking to the TME. In addition to these pathways, other crucial pathways, such as STAT3, NF-κB, and PI3K, play a crucial role in influencing TAM activities, determining whether they promote or suppress tumor growth [102].

CSF-1R is recently recognized for its role in cancer cell proliferation in solid tumors, through cell cycle downstream signaling pathways, and is supported by the TGF-B immune suppression mechanism [103]. TAMs are recruited into the TME through the CSF-1/CSF-1R axis, which guides monocyte differentiation into macrophages that typically exhibit an M2-like immunosuppressive phenotype [20,104,105]. This polarization is driven by CSF-1R signaling, which enhances the secretion of immunosuppressive cytokines such as IL-10 and TGF-β, supporting tumor growth by inhibiting cytotoxic T cell activity and promoting regulatory T cell expansion. These M2-like TAMs contribute to tumor angiogenesis, invasion, and metastasis through the release of vascular endothelial growth factor (VEGF) and matrix metalloproteinases (MMPs) [15,66].

Recent clinical trials have investigated CSF-1R as a target to modulate TAM activity. Emactuzumab and pexidartinib, for example, have demonstrated the capacity to either eliminate TAMs or convert them to a pro-inflammatory phenotype. Phase 1/2 trials showed that emactuzumab reduced TAMs in solid tumors and increased the ratio of CD8+ to CD4+ T cells [106,107]. Combination therapies using BLZ945 and other CSF-1R inhibitors are showing promise in boosting anti-tumor immunity. Clinical trial results, however, are mixed, with many showing stable disease or partial responses instead of significant shrinkage of tumors [15,108].

### 5.3. Side Effects and Safety

Systemic TAM depletion can have unintended consequences, including impaired immune function, autoimmune reactions, and increased susceptibility to infections. Localized delivery of anti-TAM therapy can enhance therapeutic efficacy without systemic side effects [11].

### 5.4. Future Directions

Employing advanced technologies like single-cell RNA sequencing, mass cytometry, and spatial transcriptomics provide detailed profiling of TAMs and their subpopulations in HCC. These methods can uncover the specific markers and functional states of TAMs. [109]. One proposed future direction is to explore the suppression of TAMs, as it was discovered that TAMs have exhaustion markers, such as TIM-3, TIGIT, LAG-3, and PD-1 [110,111,112,113]. M2 macrophages strongly express TIGIT, which reduces the release of TNF-α, IL-β, and IL-12, inhibiting macrophage-induced cytotoxicity [114]. The TIGIT (T-cell immunoreceptor with Ig and ITIM domains) molecule is known to bind to CD155 and CD112 in order to create an immune suppression mechanism [115]. Blocking TIGIT by disrupting its binding to CD155 or CD112 halts the immune suppression mechanism in the TME. M6223 and ociperlimab (OCI) are engineered antibodies that bind to TIGIT on immune cells, preventing its interaction with the ligand.

TIGIT has shown potential in enhancing anti-tumor immunity in the treatment of HCC [116]. Targeting the TIGIT pathway can lead to improved clinical outcomes, such as tumor regression and longer survival rates; however, patients may experience adverse effects, including immune-related events like rash, fatigue, and, in some cases, more severe reactions such as pneumonitis or liver toxicity [117,118,119,120].

Early clinical trials have shown that combining TIGIT inhibitors with other immunotherapies, such as PD-1/PD-L1 blockers, can lead to synergistic effects, resulting in increased tumor regression and prolonged survival. This innovative strategy represents a significant advancement for those HCC patients who may not respond adequately to traditional treatments [121,122].

Bi-specific engagers, which combine the single-chain variable fragment (scFv) of a humanized antibody against a cancer antigen with the scFv of an immune cell-specific antibody, are currently being studied in preclinical trials for HCC therapy [101].

Using bi-specific engagers has the advantage of specifically targeting and depleting immune suppressive TAMs, which can contribute to a safer systemic therapy regimen [102]. However, this particular approach has not been studied up until this point.

## 6. Conclusions

Controlling TAMs by reprogramming, inhibiting, or depleting them represents a promising approach to modifying the TME in hepatocellular carcinoma. Current strategies include the use of monoclonal antibodies, small molecule inhibitors, cytotoxic agents, and combination therapies, all of which have shown potential in preclinical and clinical studies. However, challenges such as TAMs’ heterogeneity, resistance mechanisms, and safety concerns still need to be addressed. The depletion of tumor-associated macrophages offers a promising strategy to modify the tumor microenvironment, reduce immune suppression, enhance both local and systemic immune responses in cancer, and improve the effectiveness of existing and emerging therapies. Continued research and clinical trials will be helpful in translating these strategies into improved outcomes for cancer patients.
